# Genome-wide identification, structural characterization and gene expression analysis of the *LEA_2* gene family in faba bean (*Vicia Faba* L.)

**DOI:** 10.1186/s12870-025-07671-8

**Published:** 2025-11-28

**Authors:** Yu Yin, Xinwei Wang, Haixia Ma, Qinyuan Chen, Xin Tang, Xuexia Wu, Shuo Feng

**Affiliations:** State Key Laboratory of Plateau Ecology and Agriculture, Qinghai University, Xining, 810016 People’s Republic of China

**Keywords:** Faba bean, *LEA_2* gene family, Genome-wide analysis, Gene duplication, Abiotic stress, Phytohormone response

## Abstract

**Background:**

Late Embryogenesis Abundant Protein 2 (LEA_2) plays critical roles in plant abiotic stress adaptation and developmental regulation. Faba bean (*Vicia faba* L.) is an important global crop, but its productivity is severely limited by abiotic stress. However, the *LEA_2* gene family, which is critical for stress adaptation, remains poorly characterized in this species.

**Results:**

In this study, we performed the first genome-wide identification of *LEA_2* genes in faba bean, identifying 41 members (*VfLEA_2−1*–*VfLEA_2–41*) phylogenetically classified into seven subfamilies (Groups 1–7). Chromosomal distribution analysis localized 40 *VfLEA_2* genes across seven chromosomes, while *VfLEA_2–41* resided on a contig. Tandem (4 pairs) and segmental (8 pairs) duplication events were identified as key drivers of family expansion, with collinearity analysis indicating closest evolutionary relationships to *Trifolium repens* (white clover) among six legume species. Expression profiling demonstrated tissue-specific patterns, with *VfLEA_2–4/VfLEA_2–5* (pods), *VfLEA_2–15/VfLEA_2–38* (seeds), and *VfLEA_2–21/VfLEA_2–32* (stems) showing preferential expression during development. Furthermore, *VfLEA_2* genes exhibited dynamic responses to ABA, MeJA, SA, and abiotic stresses (drought, cold, salt), with rapid induction (3–6 h post-treatment) and stress-specific expression kinetics. These findings systematically elucidate the molecular evolution, structural features and functional differentiation of *VfLEA_2* genes, providing a foundation for leveraging these candidates in faba bean breeding to enhance stress resilience and yield stability.

**Conclusions:**

This study represents the first investigation focusing on an important crop species, addressing practical challenges to its survival, and targeting crucial proteins influencing plant growth. By integrating bioinformatics with molecular biology, we systematically identified the *LEA_2* gene family in faba bean and conducted in-depth analyses of their physicochemical properties, evolutionary relationships, and expression patterns. This work establishes a theoretical foundation for future research on the functional roles of faba bean *LEA_2* genes in plant growth, development, and environmental adaptation.

**Supplementary Information:**

The online version contains supplementary material available at 10.1186/s12870-025-07671-8.

## Background

Abiotic stress (such as drought, salinity and extreme weather) is an environmental condition that can impact plant morphology, physiology and biochemical characteristics [1]. Due to its adverse effects on plant growth, it can lead to a reduction in crop productivity. In response to the challenge of abiotic stress, plants have developed a series of mechanisms at the molecular, physiological and biochemical levels. For example, transcription factors and functional stress proteins can regulate downstream signaling pathways, ultimately resulting in physiological responses to stress [2]. On the other hand, functional stress proteins in plants, such as Late Embryogenesis Abundant (LEA) proteins, can scavenge reactive oxygen species (ROS) in cells to protect macromolecules and alleviate damage caused by abiotic stress [3].

LEA proteins were first identified during late embryogenesis in cotton seeds [4, 5]. Current research identifies LEA proteins as functional stress proteins involved in cellular structure protection and macromolecule stabilization under abiotic stress conditions [2, 6]. LEA proteins are induced by chilling stress, osmotic stress and phytohormones [7, 8]. LEA proteins constitute a large protein family ubiquitously distributed in plants. Based on amino acid sequences of eight conserved PFAM domains, LEA proteins are classified into nine families (LEA_1-LEA_6, Dehydrin, SMP and AtM) [9]. The LEA_2 family exhibits distinct characteristics from other LEA proteins. Its secondary structure contains fewer random coils and is enriched in β-sheets compared to other LEA proteins. Many LEA_2 protein sequences harbor one or multiple copies of the Water stress and Hypersensitive response domain (WHy). This domain displays moderate conservation and comprises approximately 100 amino acid residues. The WHy domain core sequence typically contains the NPN/Y tripeptide motif [10]. To date, the functional role of the WHy domain remains incompletely characterized. Published evidence suggests the WHy domain may confer protection against protein denaturation [11, 12]. The stress tolerance enhancement conferred by LEA_2 proteins is potentially attributable to the WHy domain.

Edible legumes constitute a vital component of human diets, representing one of the earliest cultivated crops and serving as staple foods across numerous global cultures [13]. Faba bean is a multifunctional crop that can be used for both food and feed [14]. Faba beans contain high dietary fiber and bioactive compound content, coupled with low saturated fat, carbohydrates with a low glycemic index (GI) and cholesterol-free characteristics. These attributes contribute to reduced insulin secretion and prevention of chronic diseases including obesity, cancer, diabetes and cardiovascular disorders, thereby promoting health and longevity [15]. Beyond human nutrition, faba beans play a significant role in livestock production by providing protein and energy resources, with dietary incorporation reducing feed costs and ensuring stable supply [16]. Under abiotic stress, plants evolve adaptive mechanisms through gene family expansion via tandem duplication. As the largest *LEA* family, *LEA_2* exhibits substantial membership. During its expansion through tandem duplication events, functional diversification has occurred within the *LEA_2* family. This evolutionary process explains the observed functional diversity among *LEA_2* members under stress conditions [17].

Recent research on legume *LEA* gene families has produced key discoveries. The soybean gene *GmLEA4_19* appears to function in plant height and drought tolerance regulation [18]. Additionally, chickpea *CarLEA4* may participate in various developmental processes and abiotic stress responses [19]. Furthermore, in pea, LEA proteins are relatively abundant, although they represent the least studied component of seed proteins [20]. Although numerous studies have been conducted on LEA proteins across diverse legumes, the *LEA_2* gene family in faba bean remains poorly characterized. In this study, we performed a genome-wide identification and systematic analysis of *LEA_2* genes in faba bean through comprehensive bioinformatics strategies. The *LEA_2* gene family members were identified based on conserved domain screening and phylogenetic classification. Further analyses included gene structure organization, chromosomal localization, cis-acting regulatory elements in promoter regions and evolutionary relationships with *LEA_2* homologs from other representative plant species. This work provides the first comprehensive characterization of the *LEA_2* gene family in faba bean, offering foundational insights for future functional investigations and crop improvement strategies targeting abiotic stress tolerance.

## Results

### Genome-wide identification of *LEA_2* gene family members in faba bean

Through comparative analysis with *Arabidopsis thaliana* using BLAST tools, 41 *LEA_2* gene family members in faba bean were identified (Supplementary Table 1). These genes were systematically designated as *VfLEA_2 − 1* to *VfLEA_2–41* according to their chromosomal localization, reflecting their sequential positions on the chromosomes. Subsequently, comprehensive physicochemical characterization of the 41 VfLEA_2 proteins was performed, including analyses of molecular weight, protein isoelectric point (pI) and subcellular localization.

Among the 41 VfLEA_2 proteins, VfLEA_2–17 was the longest (379 amino acids) and possessed the highest molecular weight (42.52 kD), whereas VfLEA_2–34 was the shortest (139 amino acids) and had the lowest molecular weight (16.06 kD). The isoelectric points (pI) of all VfLEA_2 proteins in this study ranged from 5.35 to 10.35. Most proteins (approximately 87.8%) exhibited a pI greater than 7. As observed in previous studies, this indicated a net positive surface charge at neutral pH. This property was conferred by a relatively high content of basic amino acids [21, 22]. Therefore, most VfLEA proteins were rich in basic amino acids. The subcellular localization of *VfLEA_2* genes was predicted using WoLF PSORT, revealing distinct compartmentalization patterns. The analysis demonstrated that *VfLEA_2–29* was targeted to the mitochondrion, and *VfLEA_2–23* was targeted to the vacuole, while 15 genes (*VfLEA_2−1*, *VfLEA_2–2*, *VfLEA_2–8*, *VfLEA_2–10*, *VfLEA_2–12*, *VfLEA_2–13*, *VfLEA_2–18*, *VfLEA_2–24*, *VfLEA_2–25*, *VfLEA_2–26*, *VfLEA_2–27*, *VfLEA_2–33*, *VfLEA_2–34*, *VfLEA_2–36*, *VfLEA_2–37*) were localized to the cytoplasm. 14 genes (*VfLEA_2–3*, *VfLEA_2–6*, *VfLEA_2–7*, *VfLEA_2–14*, *VfLEA_2–16*, *VfLEA_2–17*, *VfLEA_2–19*, *VfLEA_2–20*, *VfLEA_2–22*, *VfLEA_2–28*, *VfLEA_2–31*, *VfLEA_2–38*, *VfLEA_2–39*, *VfLEA_2–40*) were predicted to reside in the chloroplast, and 3 genes (*VfLEA_2–9*, *VfLEA_2–15*, *VfLEA_2–41*) were associated with the cell wall. Additionally, 2 genes (*VfLEA_2–11*, *VfLEA_2–32*) were assigned to the Golgi apparatus, and 2 others (*VfLEA_2–30*, *VfLEA_2–35*) to the peroxisome. The endoplasmic reticulum and nucleus were predicted to contain 3 genes (*VfLEA_2–4*, *VfLEA_2–5*, *VfLEA_2–21*) (Supplementary Table 1).

### Phylogenetic analysis and classification of VfLEA_2 proteins

**Fig. 1:**
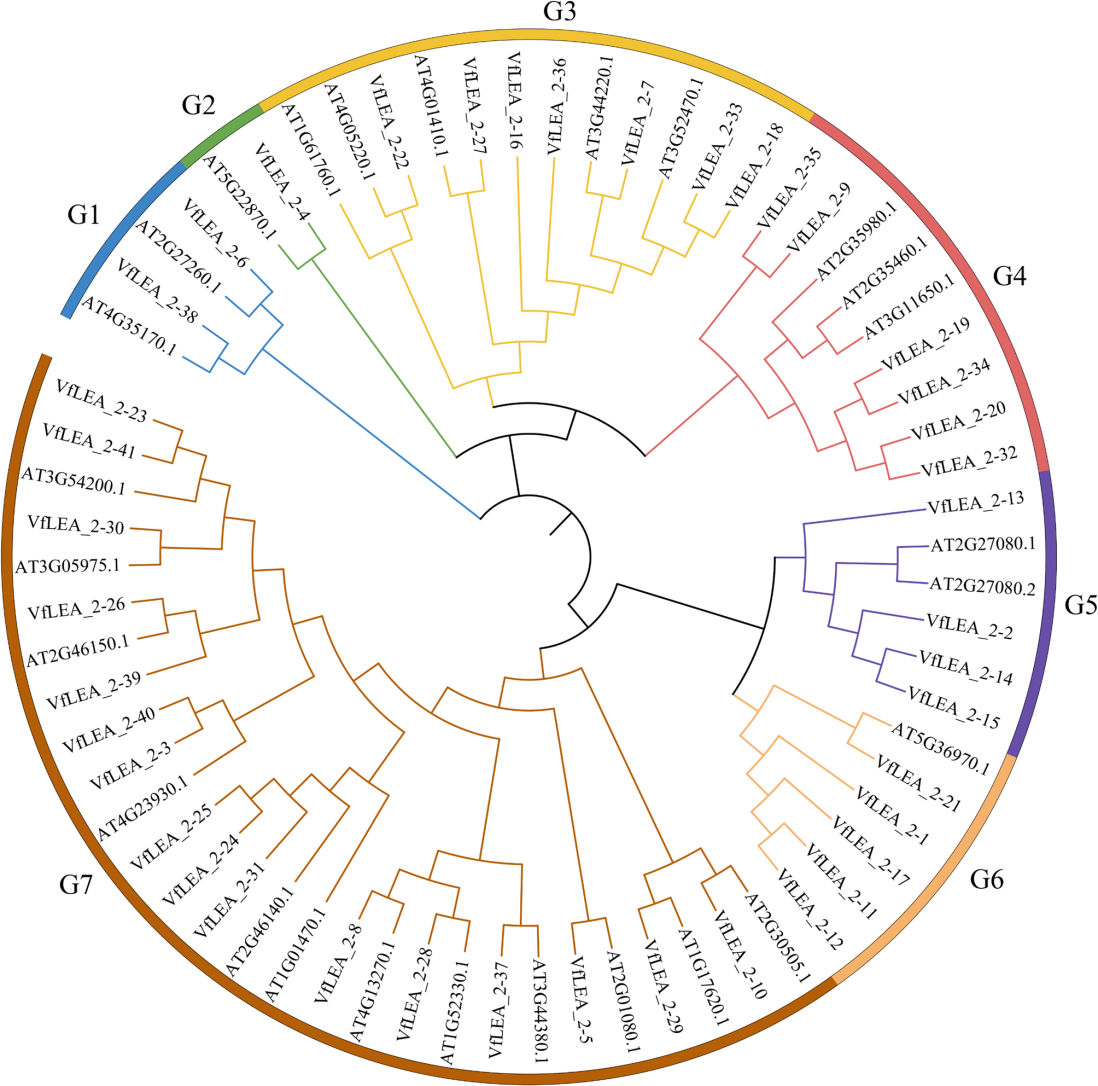
Phylogenetic tree of the relationships between the LEA_2 proteins of faba bean and *Arabidopsi thaliana*, G1, G2, G3, G4, G5, G6 and G7 represent the different subfamilies

A maximum-likelihood phylogenetic tree was constructed using MEGA software with 41 identified VfLEA_2 and 26 *Arabidopsis thaliana* LEA_2 protein sequences, based on their full-length amino acid alignments. The classification followed the established *Arabidopsi thaliana* LEA_2 nomenclature, resolving the VfLEA_2 family into seven subfamilies: Group 1, 2, 3, 4, 5, 6, and 7 (Fig. 1; Bootstrap = 1,000 replicates). Subfamily 7 contained the largest clade (16 VfLEA_2), followed by group 3 (7 VfLEA_2). And then group 1 has 2 VfLEA_2, group 2 has 1 VfLEA_2, group 4 has 6 VfLEA_2, group 5 has 4 VfLEA_2 and group 6 has 5 VfLEA_2.

### Comprehensive analysis of conserved motifs, gene structure and cis-acting elements in *VfLEA_2* genes

**Fig. 2:**
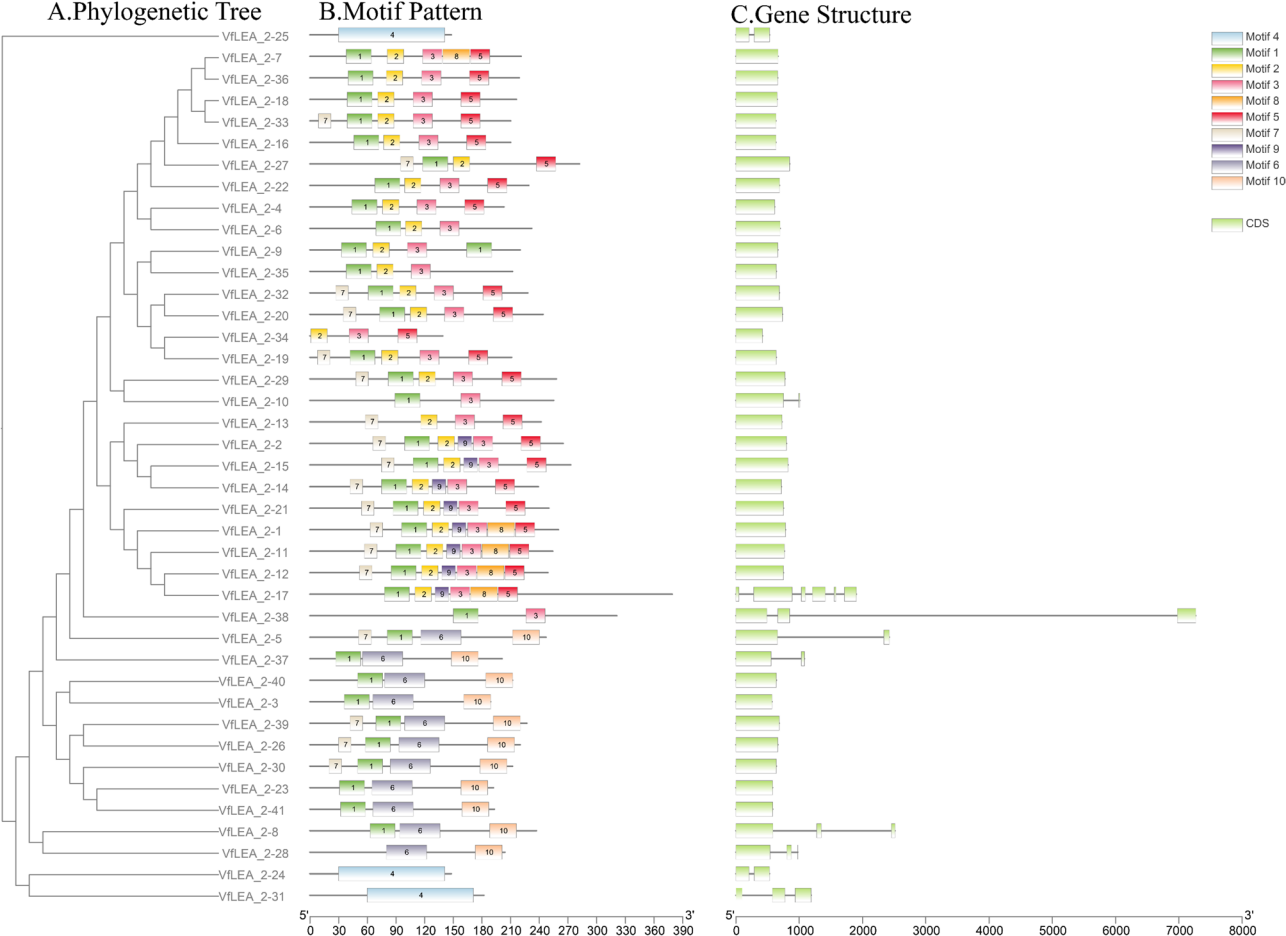
Phylogenetic tree, motif pattern and gene structure of VfLEA_2 proteins. **A** The phylogenetic tree was constructed using the full-length sequences of VfLEA_2 proteins with 1000 replicates on each node. **B** The amino acid motifs (numbered 1–10) in VfLEA_2 proteins are displayed in ten colored boxes, and black lines indicate amino acid length. **C** Exon-intron structures of *VfLEA_2* genes. Green bar: exons; black lines: introns

Comparative analysis of exon-intron configurations revealed that all 41 *VfLEA_2* genes contained variable exon numbers. To investigate evolutionary diversification within the *VfLEA_2* gene family, MEME suite was employed to analyze conserved motifs across 41 VfLEA_2 proteins, identifying 10 distinct motifs (designated Motif 1–10). Analysis of motif composition revealed that the majority of VfLEA_2 proteins contained motifs 1, 2, 3, and 5. Strikingly, only VfLEA_2–24, VfLEA_2–25 and VfLEA_2–31 were found to contain motif 4 (Fig. 2B, Supplementary Table 2).

Gene structure is a pivotal determinant of gene function. In this study, we analyzed the exon - intron structure and conserved motifs of *VfLEA_2* genes in faba bean. Notably, most *VfLEA_2* genes are intron-less or contain a single intron. However, there are exceptions: *VfLEA_2–8*, *VfLEA_2–28*, *VfLEA_2–31*, and *VfLEA_2–38* each have two introns, *VfLEA_2–17* contains five introns. Overall, *VfLEA_2* genes within the same group exhibit similar exon-intron structures (Fig. 2C), which corroborates their group classification and phylogenetic relationships.

**Fig. 3:**
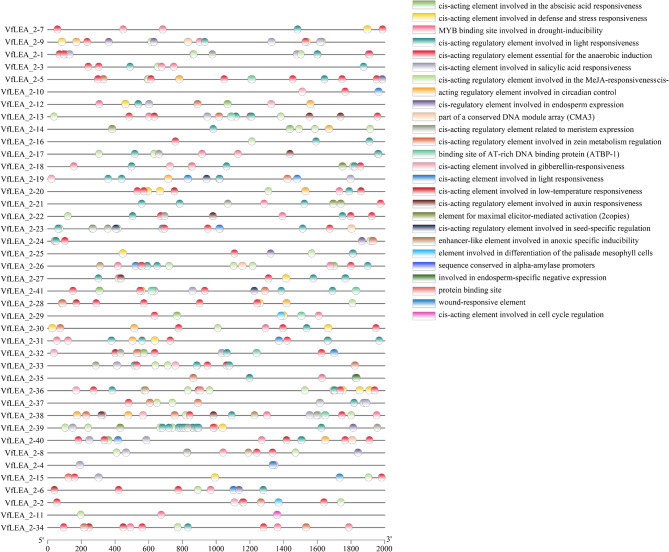
The promoter cis-acting elements of *VfLEA_2* genes are shown in colored boxes

The identified elements were categorized into functional groups, including hormone responsiveness (e.g., ABA, IAA, GA, MeJA and SA), low-temperature response, light response, circadian rhythm regulation and anaerobic induction. Among stress- and hormone-associated cis-acting elements, MeJA-responsive elements (118 occurrences), light-responsive elements (97 occurrences) and abscisic acid-responsive (ABA, 82 occurrences) exhibited the most abundant distribution (Fig. 3, Supplementary Table 3).

### Chromosomal distribution and duplication analysis of *VfLEA_2* genes

**Fig. 4:**
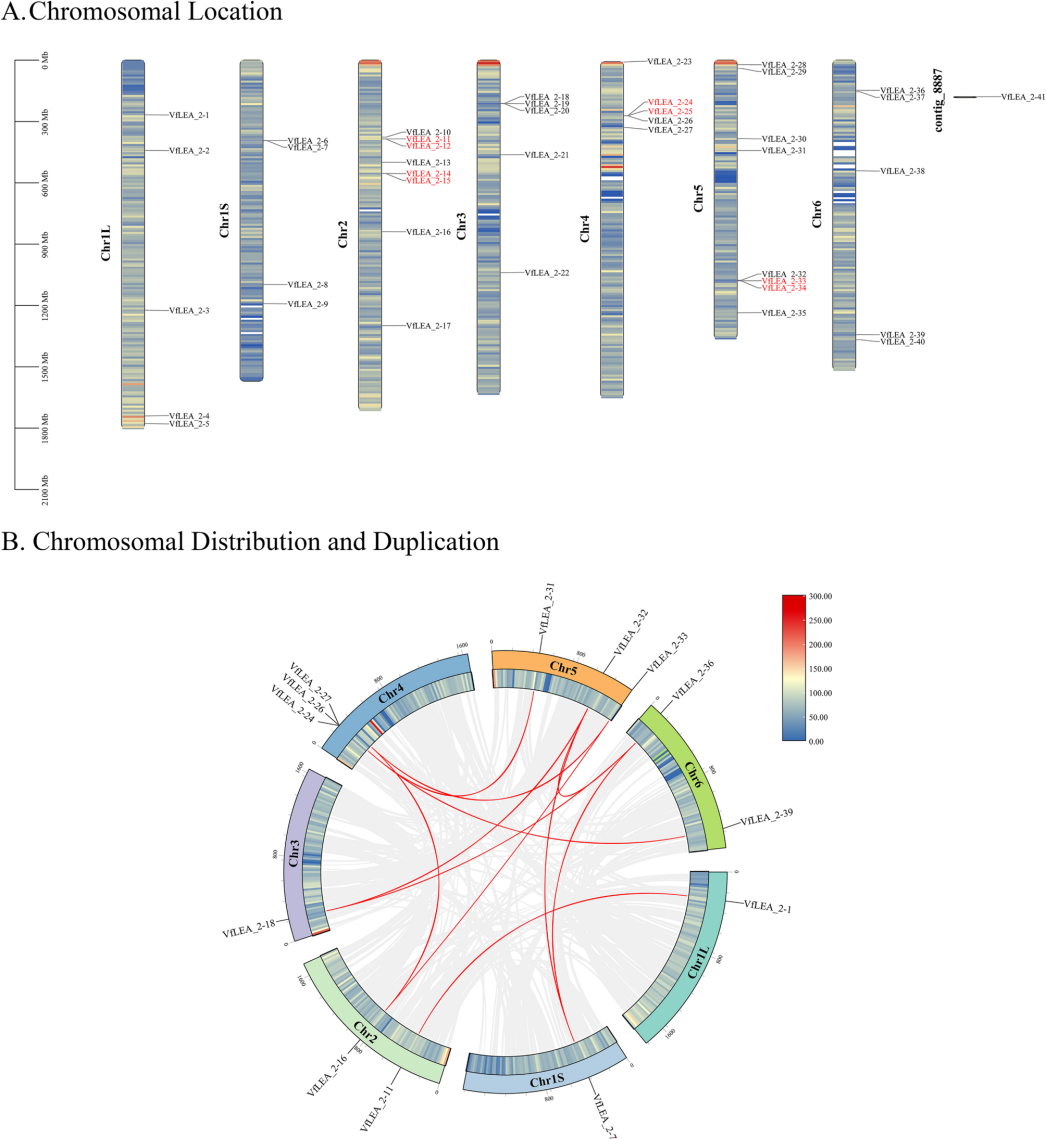
Chromosomal location and Chromosomal distribution and duplication of *VfLEA_2* genes. **A** Chromosomal location of *VfLEA_2* genes. Colored bars indicate faba bean chromosomes; Chr1L-6 denote different chromosomes; red fonts mark tandemly duplicated genes; and the 0–2100 Mb scale shows chromosome length. **B** Chromosomal distribution and duplication of *VfLEA_2* genes. Gray lines represent collinear regions in the faba bean genome, red lines indicate duplicated *LEA_2* gene pairs, and chromosome numbers are labeled within each chromosome

The 40 *VfLEA_2* genes were unevenly distributed across seven chromosomes, with members of the same family exhibiting a random distribution pattern. Analysis revealed that Chr1L contained 5 genes (*VfLEA_2-1-VfLEA_2–5*, 12.2%), Chr1S contained 4 genes (*VfLEA_2-6-VfLEA_2–9*, 9.8%), Chr2 contained 8 genes (*VfLEA_2-10-VfLEA_2–17*, 19.5%), Chr3 contained 5 genes (*VfLEA_2-18-VfLEA_2–22*, 12.2%), Chr4 contained 5 genes (*VfLEA_2-23-VfLEA_2–27*, 12.2%), Chr5 contained 8 genes (*VfLEA_2-28-VfLEA_2–35*, 19.5%), and Chr6 contained 5 genes (*VfLEA_2-36-VfLEA_2–40*, 12.2%). Notably, *VfLEA_2–41* was located on a contig (Fig. 4A, Supplementary Table 1).

Tandem duplication events were defined as chromosomal regions containing two or more paralogous genes within a 200 kb span [23]. In this study, eight *VfLEA_2* genes (*VfLEA_2–11/VfLEA_2–12*, *VfLEA_2–14/VfLEA_2–15*, *VfLEA_2–24/VfLEA_2–25* and *VfLEA_2–33/VfLEA_2–34*) were identified as tandem duplicates localized on Chr2, Chr4 and Chr5 (Supplementary Table 4, Fig. 4A). Phylogenetic analysis demonstrated that all tandemly duplicated genes were clustered within their respective subfamilies. For example, the homologous gene pairs *VfLEA_2–11/VfLEA_2–12* (Group 6) and *VfLEA_2–14/VfLEA_2–15* (Group 5) originated from tandem duplication events (Supplementary Table 4, Figs. 1 and 4A).

Through comprehensive phylogenetic and collinearity analyses employing Blastp alignment and MCScanX algorithms on 41 faba bean *VfLEA_2* genes, we identified 16 syntenic sites with conserved evolutionary relationships and detected 8 segmental duplication events: *VfLEA_2 − 1/VfLEA_2–11*, *VfLEA_2–7/VfLEA_2–32*, *VfLEA_2–7/VfLEA_2–36*, *VfLEA_2–16/VfLEA_2–27*, *VfLEA_2–18/VfLEA_2–33*, *VfLEA_2–18/VfLEA_2–36*, *VfLEA_2–24/VfLEA_2–31*, *VfLEA_2–26/VfLEA_2–39*. Notably, three *VfLEA_2* genes, *VfLEA_2–7*, *VfLEA_2–18* and *VfLEA_2–36* exhibited segmental duplications with two distinct paralogs each. Specifically, *VfLEA_2–7* was duplicated with both *VfLEA_2–32* and *VfLEA_2–36*, *VfLEA_2–18* showed duplication events with *VfLEA_2–33* and *VfLEA_2–36*, and *VfLEA_2–36* was duplicated with both *VfLEA_2–7* and *VfLEA_2–18* (Fig. 4B, Supplementary Table 5).

### Collinearity analysis of the *VfLEA_2* and *LEA_2* genes in different species

**Fig. 5:**
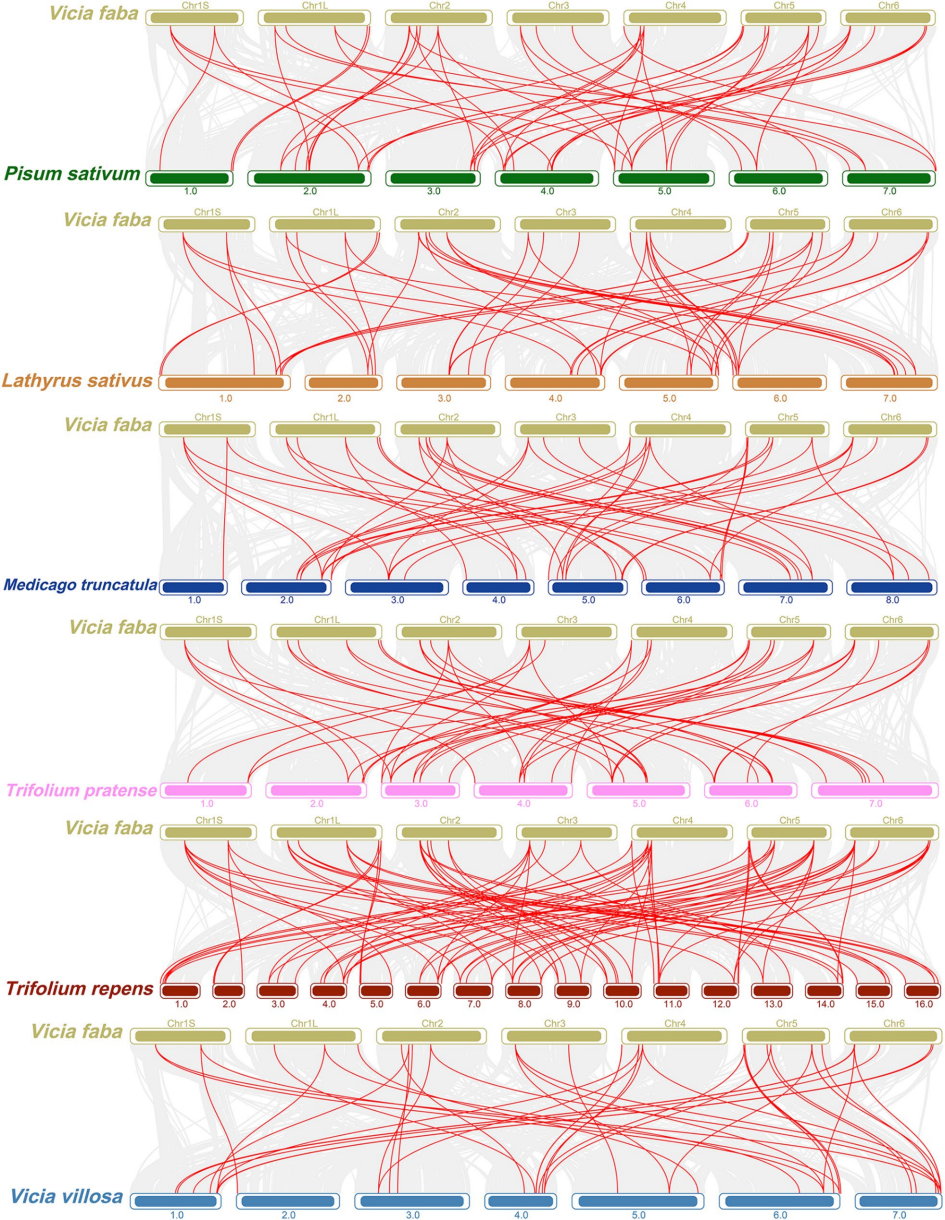
Collinearity analysis of *LEA_2* genes between faba bean and six other plants (*Pisum sativum*, *Lathyrus sativus*, *Medicago truncatula*, *Trifolium pratense*, *Trifolium repens* and *Vicia villosa*). Gray lines between faba bean and the other plants represent collinear blocks in the wide regions of the genomes, while red lines indicate the orthologous relationships of *LEA_2* genes

To investigate the evolutionary dynamics of *LEA_2* genes in faba bean, we performed BLAST-based homology searches and constructed syntenic maps between faba bean and six legume species: pea (*Pisum sativum*), grass pea (*Lathyrus sativus*), barrel medic (*Medicago truncatula*), red clover (*Trifolium pratense*), white clover (*Trifolium repens*) and hairy vetch (*Vicia villosa*). Interspecific collinearity analysis identified 55, 57, 53, 57, 100 and 47 homologous gene pairs between faba bean and each species. Notably, faba bean exhibited the most pronounced syntenic contribution with T*rifolium repens* (Fig. 5, Supplementary Table 6).

### Expression patterns of ***VfLEA_2*** genes in different plant tissues and grain development

**Fig. 6:**
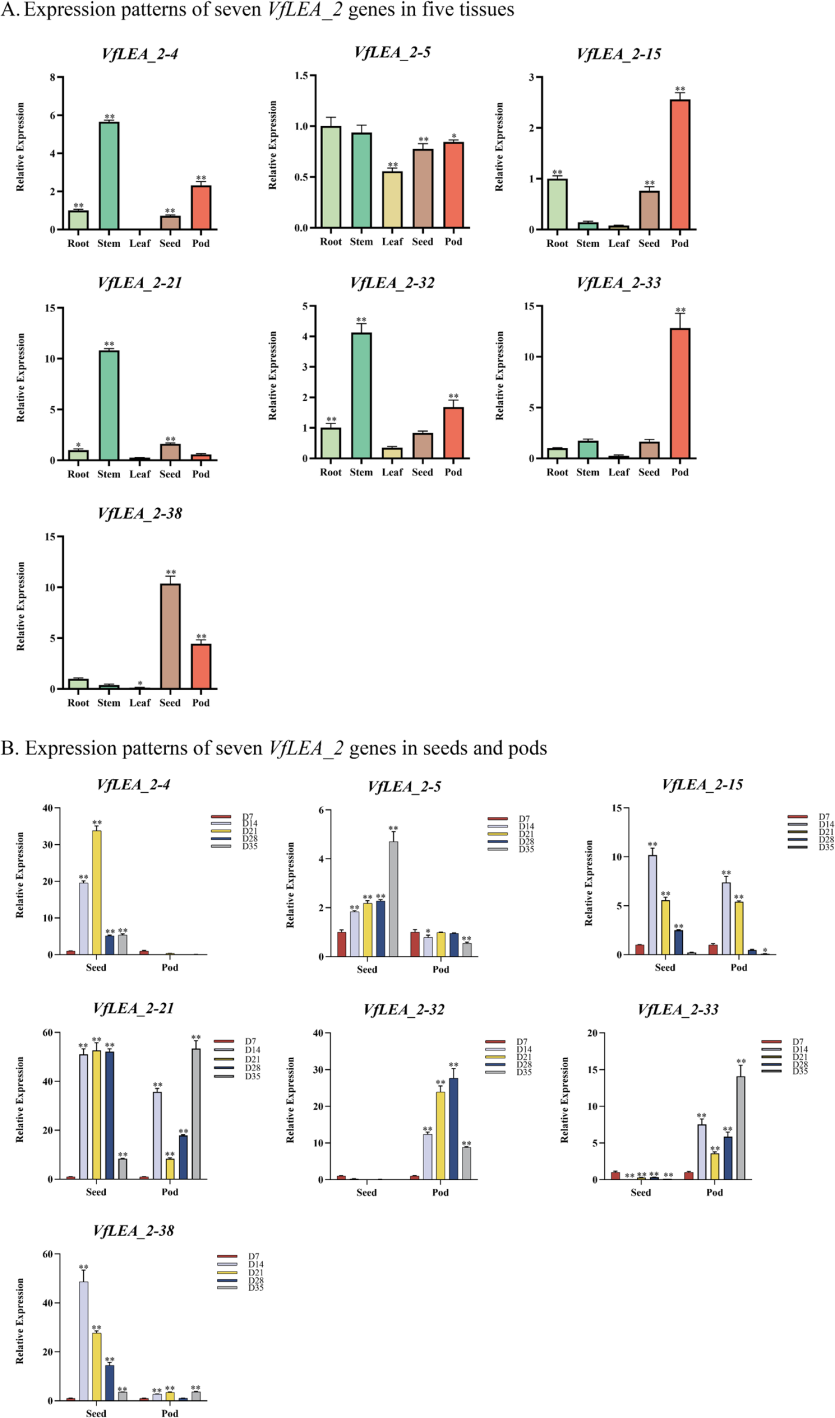
Expression analysis of *VfLEA_2* genes in faba bean. **A** Expression patterns of seven *VfLEA_2* genes in five tissues (roots, stems, leaves, seeds, and pods) at 21 days. Using the expression level at 0D as the control. The * indicates significant differences by t-test (*p < 0.05, **p < 0.01). **B** Expression patterns of seven *VfLEA_2* genes in seeds and pods at different time points during grain filling. Using the expression level at 0D as the control. The * indicates significant differences by t-test (*p < 0.05, **p < 0.01)

The expression patterns of 7 *VfLEA_2* genes in faba bean were analyzed through qRT-PCR. In the tissues (root, stem, leaf, seed, pod) at 21 days, *VfLEA_2–5*,* VfLEA_2–15* and *VfLEA_2–33* showed higher expression in pods, while *VfLEA_2–4*, *VfLEA_2–21* and *VfLEA_2–32* in stems, *VfLEA_2–38* in seeds (Fig. 6A, Supplementary Table 7).

During faba bean grain filling, the expression of *VfLEA_2* genes in seeds and pods was examined. *VfLEA_2–4* had higher expression in seeds at D14 and D21, *VfLEA_2–5* in seeds at D28 and D35, *VfLEA_2–15* in seeds at D14 and D21, *VfLEA_2–21* in pods at D35, *VfLEA_2–32* in pods at D21 and D28, *VfLEA_2–33* in pods at D14 and D35, and *VfLEA_2–38* in seeds at D14 and D21 (Fig. 6B).

**Fig. 7:**
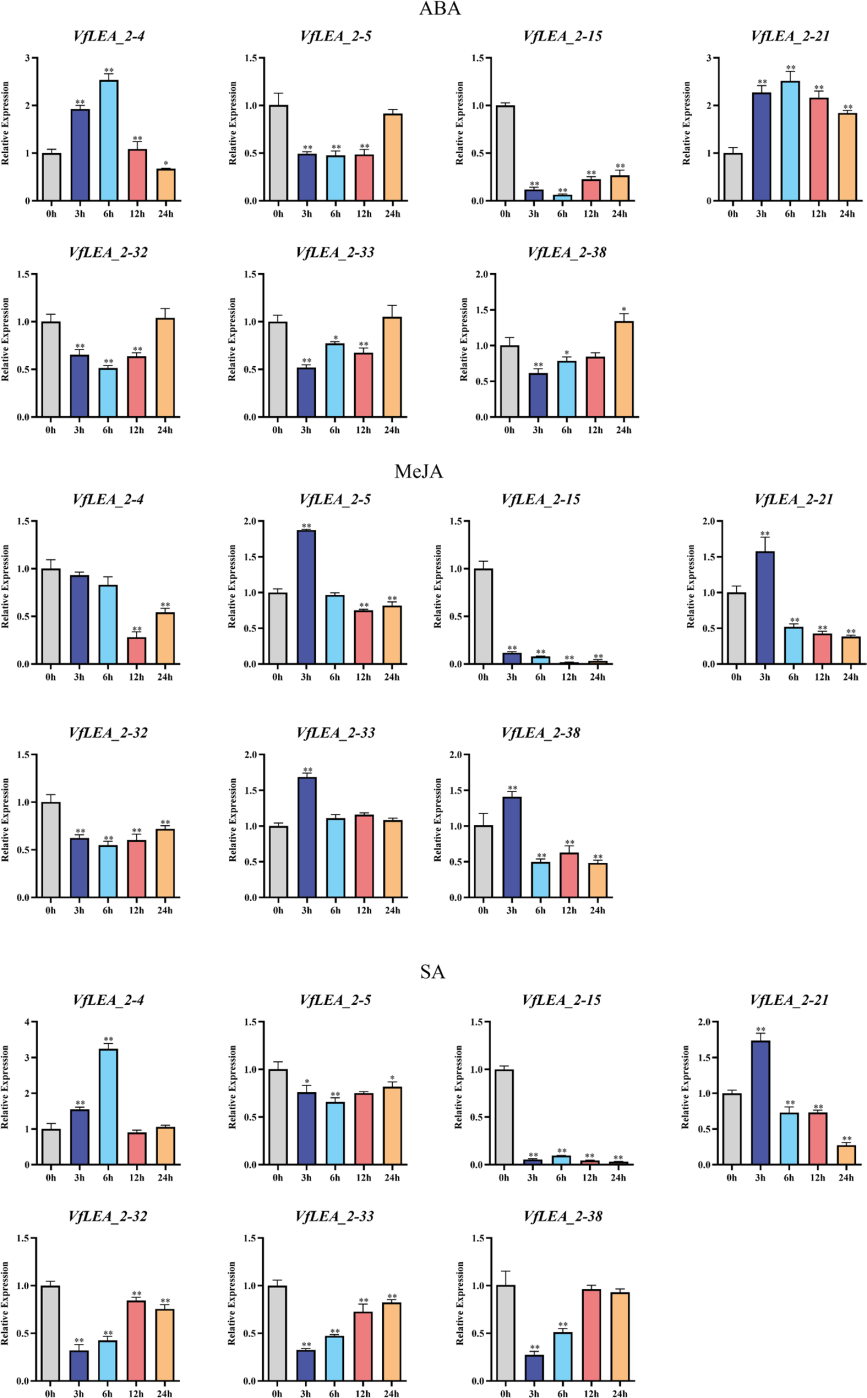
Expression patterns of 7 *VfLEA_2* genes in faba bean seedlings after 3 hormone treatments (ABA, MeJA, SA). Using the expression level at 0 h as the control. The * indicates significant differences by t-test (*p < 0.05, **p < 0.01)

After ABA, MeJA and SA hormone treatments, the expression patterns of *VfLEA_2* genes in faba bean seedlings were assessed. Under ABA treatment, *VfLEA_2–4* showed increased expression at 3 h and 6 h, *VfLEA_2–15* at 12 h and 24 h, *VfLEA_2–21* at 3 h and 6 h, *VfLEA_2–32* at 12 h and 24 h, *VfLEA_2–33* at 6 h and *VfLEA_2–38* at 24 h. MeJA treatment led to increased expression of *VfLEA_2–5* at 3 h, *VfLEA_2–21* at 3 h, *VfLEA_2–33* at 3 h, and *VfLEA_2–38* at 3 h. The expression level of *VfLEA_2–15* exhibited a progressive and sustained decrease over time. SA treatment resulted in increased expression of *VfLEA_2–4* at 6 h, *VfLEA_2–21* at 3 h, *VfLEA_2–32* at 12 h (Fig. 7).

**Fig. 8:**
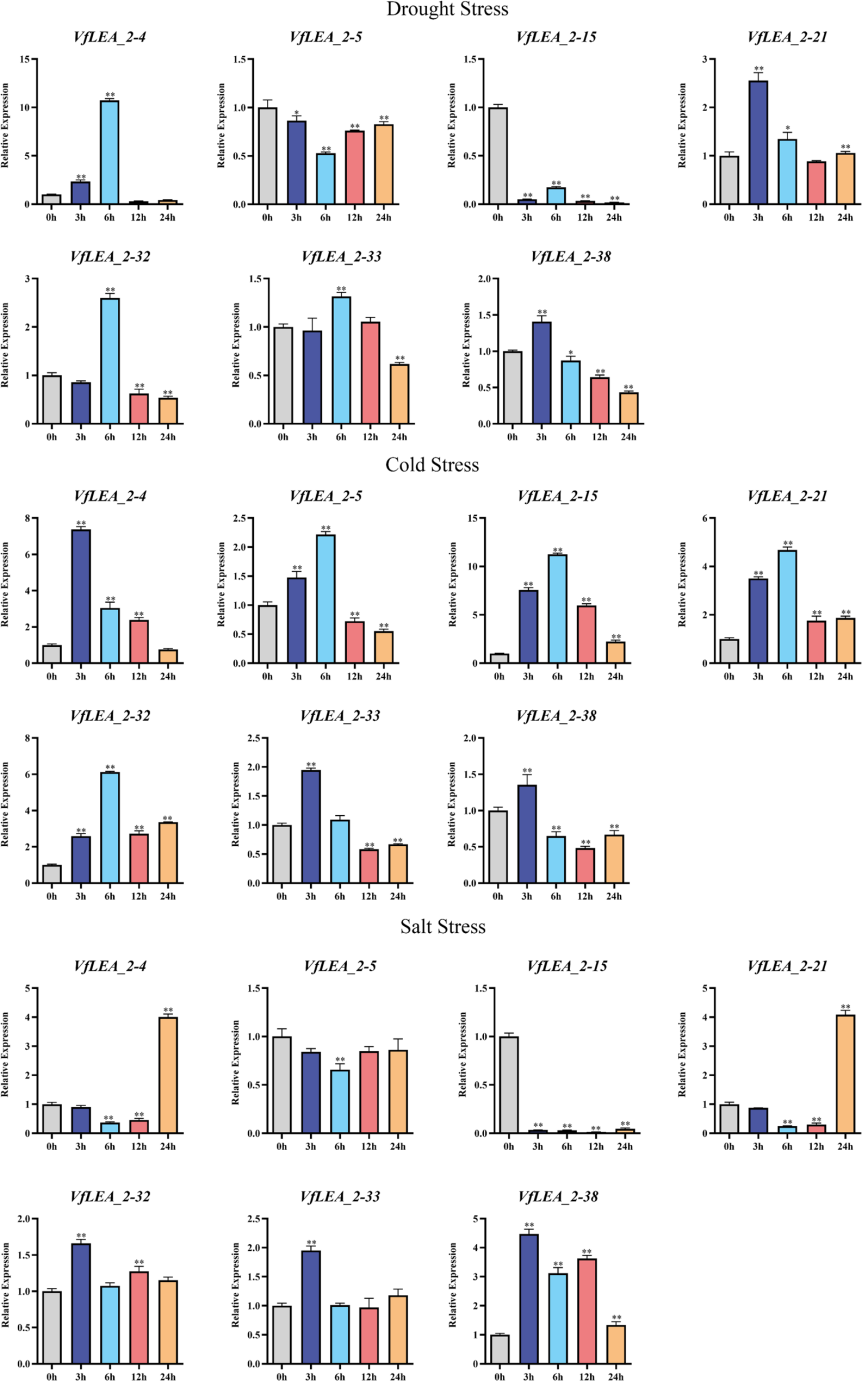
Expression patterns of 7 *VfLEA_2* genes in faba bean seedlings after 3 stress treatments (Drought Stress, Cold Stress, Salt Stress). Using the expression level at 0 h as the control. The * indicates significant differences by t-test (*p < 0.05, **p < 0.01)

Under drought and cold stress conditions, the expression patterns of *VfLEA_2* genes were analyzed. Drought stress led to increased expression of *VfLEA_2–4* at 6 h, *VfLEA_2–5* at 12 and 24 h, *VfLEA_2–21* at 3 h, *VfLEA_2–32* at 6 h, *VfLEA_2–33* at 6 h, and *VfLEA_2–38* at 3 h. *VfLEA_2–15* exhibited consistently low transcript levels, whereas *VfLEA_2–38* showed a progressive and sustained decline in expression over time. Cold stress resulted in increased expression of *VfLEA_2–4* at 3 h, *VfLEA_2–5* at 3 h and 6 h, *VfLEA_2–15* at 3 h and 6 h, *VfLEA_2–21* at 3 h and 6 h, *VfLEA_2–32* at 6 h, *VfLEA_2–33* at 3 h, and *VfLEA_2–38* at 3 h (Fig. 8).

After salt stress treatment, the expression patterns of *VfLEA_2* genes in faba bean seedlings were evaluated. *VfLEA_2–4* showed increased expression at 24 h, *VfLEA_2–21* at 24 h, *VfLEA_2–32* at 3 h, *VfLEA_2–33* at 3 h, and *VfLEA_2–38* at 3 h and 12 h, *VfLEA_2–15* maintained consistently low transcript abundance throughout the experimental period (Fig. 8).

## Discussion

LEA_2 proteins are DNA-binding proteins predominantly found in plants that can respond to a variety of abiotic stresses. The phylogenetic classification of 41 VfLEA_2 proteins into seven subfamilies (Groups 1–7) aligns with the established nomenclature for *Arabidopsis thaliana* LEA_2 proteins [24, 25] (Fig. 1, Supplementary Table 1), suggesting evolutionary conservation of functional domains across divergent plant lineages. The expansion of Group 7 *VfLEA_2* genes, which includes seed maturation-associated LEAM and LEAP proteins [26], correlates with the prominence of seed desiccation tolerance mechanisms in legumes [27]. Predicted subcellular localizations of *VfLEA_2* genes highlight their functional compartmentalization. Cytoplasmic localization of 11 *VfLEA_2* genes (e.g., *VfLEA_2-1*, *VfLEA_2-26*) aligns with the role of *LEA_2* genes in stabilizing cytosolic enzymes and membranes during dehydration [28]. Chloroplast-targeted *VfLEA_2–6* and *VfLEA_2–19* may protect photosynthetic machinery, as demonstrated for *Arabidopsis thaliana AtLEA7* (CHLOROPLAST-LOCALIZED *LEA*) under oxidative stress [29]. The mitochondrial localization of *VfLEA_2–29* parallels the protective role of LEAM proteins in preserving mitochondrial membrane integrity during seed desiccation [30] (Supplementary Table 1).

The physicochemical properties of the faba bean *LEA_2* gene family, such as protein length, molecular weight (MW) and protein isoelectric point (pI) are highly variable, but its gene structure and amino acid motifs are relatively conserved. The conserved motif composition of *VfLEA_2* genes reflect functional constraints on protein structure. The prevalence of motifs 1, 2, 3, and 5 across most *VfLEA_2* genes suggests these motifs encode core structural elements critical for chaperone-like activity or membrane stabilization during desiccation [31]. The exclusive presence of motif 4 in *VfLEA_2–24*, *VfLEA_2–25* and *VfLEA_2–31* implies specialized roles for these genes, potentially in binding ions or nucleic acids- a feature observed in Group 3 LEAM proteins [32] (Fig. 2B, Supplementary Table 3). Previous studies have shown that genetic evolution leads to a low number of introns. When there are fewer introns, the time from transcription to translation shortens, enabling genes to quickly produce functional proteins in response to stress [33–35]. This feature of having few introns is also present in other stress-related gene families, such as *hsp20* [36]. In this study, as many as 87.8% of *VfLEA_2* genes contain no intron or just one intron (Fig. 2C), which further indicates that *VfLEA_2* is a stress-response family.

Tandem and segmental duplication events have played pivotal roles in *VfLEA_2* family expansion. The identification of eight tandem duplicates (e.g., *VfLEA_2–11/VfLEA_2–12*, *VfLEA_2–24/VfLEA_2–25*) (Fig. 4A, Supplementary Table 4) and eight segmental duplication pairs (e.g., *VfLEA_2–7/VfLEA_2–32*, *VfLEA_2–18/VfLEA_2–36*) (Fig. 4B, Supplementary Table 5) underscores the importance of local gene duplication and whole-genome duplication (WGD) events in shaping the legume *LEA_2* repertoire. These duplication events align with the evolutionary history of the Fabaceae family. This plant family has undergone multiple whole-genome duplication events since it diverged from *Arabidopsis thaliana* [37]. Retained duplicates often exhibit subfunctionalization or neofunctionalization [38], as evidenced by the tissue-specific expression of paralogs like *VfLEA_2–4* (stem-enriched) and *VfLEA_2–38* (seed-enriched) (Fig. 6A). Collinearity analysis between faba bean and six legume species revealed extensive synteny, with 47–100 homologous gene pairs per species (Fig. 5, Supplementary Table 6). This conservation reflects strong purifying selection on *LEA_2* genes critical for legume survival in drought-prone habitats. Notably, the higher number of syntenic loci in white clover (100) compared to faba bean (41) may stem from its recent polyploidization [39], highlighting how WGD amplifies stress gene families in polyploids.

ABRE, MYB/MYC and Sp1 cis-regulatory elements are abundantly distributed within the promoter regions of *VfLEA_2* genes (Fig. 3). These elements participate in regulating the expression of downstream genes under abiotic stress conditions [40]. The promoter analysis revealed an abundance of stress- and hormone-responsive cis-elements, including MeJA- (118), ABA- (82) and light-responsive (97) motifs. This regulatory architecture mirrors findings in soybean and chickpea, where *LEA_2* promoters are enriched in ABRE (ABA-responsive), MYB/MYC (drought-inducible), and G-box (light-regulated) elements. The high frequency of MeJA-responsive elements (CGTCA motifs) suggests crosstalk between jasmonate signaling and *LEA* gene activation-a phenomenon observed in *Arabidopsis thaliana* under osmotic stress. ABA-responsive elements (ABREs) likely mediate drought and cold induction of *VfLEA_2* genes, consistent with ABA’s central role in abiotic stress signaling [41] (Fig. 3, Supplementary Table 3). Hormonal treatments revealed nuanced regulatory networks: ABA-induced expression of *VfLEA_2–4/VfLEA_2–5* corroborates the canonical ABA-ABRE signaling pathway [42], while MeJA-responsive *VfLEA_2–32/VfLEA_2–33* may mediate cross-talk between jasmonate and osmotic stress pathways, as seen in tomato [43]. Promoter analysis of *Picea mongolica* revealed an enrichment of MeJA-responsive cis-elements, implying that these motifs confer a protective role under stress and may enhance the species’ tolerance to diverse abiotic stresses [44]. The SA-induced upregulation of *VfLEA_2–38* suggests a role in biotic stress responses, expanding the functional repertoire of *LEA_2* genes beyond abiotic stress (Fig. 3, Supplementary Table 3). Notably, the co-occurrence of light-responsive elements (e.g., G-box, Sp1) and circadian regulators (e.g., circadian control) in *VfLEA_2* promoters implies diurnal regulation of *LEA* expression, as reported in rice. This integration of photoperiodic and stress signals may optimize resource allocation during stress recovery [45] (Fig. 3, Supplementary Table 3).

The tissue-specific expression profiles of *VfLEA_2* genes reflect their roles in developmental and stress adaptation processes. The preferential expression of *VfLEA_2–5* in pods correlates with their putative function in desiccation tolerance during seed maturation- a trait critical for legume yield [46] (Fig. 6A and B). Similarly, the seed-enriched expression of *VfLEA_2–38* parallels the accumulation of *LEA_2* genes in maturing seeds of *Medicago truncatula*. Under abiotic stresses, *VfLEA_2* genes exhibited rapid induction kinetics, with peak expression at 3–6 h post-treatment for most genes (Figs. 7 and 8). This early response aligns with the role of LEA proteins as immediate protectants against cellular damage [47]. The sustained upregulation of *VfLEA_2–15* under prolonged drought (6 h) suggests their involvement in long-term acclimation, potentially through interactions with late-responsive functional stress proteins (e.g., DREB2A) (Fig. 8).

## Conclusion

This study is the first to identify 41 *LEA_2* genes in the faba bean genome. These genes were systematically classified into seven distinct subfamilies. Comprehensive analysis revealed significant differences in the physicochemical properties among VfLEA_2 members. However, their gene structures and conserved motifs remained evolutionarily conserved. Genomic duplication analysis identified four tandem and eight segmental duplication events. This indicates that both localized and large-scale duplication events contributed to the expansion of this gene family. Expression profiling demonstrated tissue-specific patterns during development. Different members showed preferential expression in roots, stems, leaves, seeds, or pods. Additionally, *VfLEA_2* genes displayed dynamic transcriptional responses to phytohormones and abiotic stresses.

Based on the above studies, we propose the following future research directions and applications: specific *VfLEA_2* members that showed significant expression under abiotic stress or phytohormone treatments are prime targets for CRISPR-Cas9-mediated gene editing [48]. Such experiments can precisely elucidate their roles in stress adaptation and help develop stress-tolerant faba bean varieties. The identified tandem and segmental duplication events reveal the evolutionary mechanisms that shaped this gene family [49]. This insight, together with expression profiling, enables the selection of candidate genes for marker-assisted breeding or transgenic approaches [50]. In summary, this work clarifies the molecular evolution and structural diversification of the *LEA_2* gene family. Its ultimate value lies in enabling functional characterization and biotechnological utilization. This study establishes a foundational resource for future functional and applied research.

## Materials and methods

### Genome-wide identification of *LEA_2* genes in faba bean

The genomic DNA, coding sequence (CDS) and protein sequence files for faba bean were retrieved from the National Center for Biotechnology Information (NCBI, https://www.ncbi.nlm.nih.gov/, NCBI BioProject Accession ID PRJEB52541). And then, the file for the Hidden Markov model (HMM) associated with the LEA_2 structural domain (PF03168) was obtained from the PFAM database (http://pfam.xfam.org/). The *LEA_2* gene sequences from *Arabidopsis thaliana*, accessible via the TAIR database (https://www.Arabidopsisthaliana.org/), were employed to compare and eliminate redundancy in the faba bean genome file. This process utilized BLASTp (e-value ≤ 1e-10, score value ≥ 100) to identify all potential VfLEA_2 proteins. The last, the conserved structural domains were investigated through the CD-Search database (https://www.ncbi.nlm.nih.gov/Structure/cdd/cdd.shtml), while the SMART tool (http://smart.embl-heidelberg.de/) was employed for the detection of potential *VfLEA_2* genes. Additionally, the physicochemical characteristics and subcellular distribution of the characterized *VfLEA_2* family members were predicted utilizing the online platforms ProtParam (https://web.expasy.org/protparam/) and WoLF PSORT (https://wolfpsort.hgc.jp/).

### LEA_2 conserved domain, gene structure and cis-acting elements analysis

To investigate the LEA_2 proteins in *Arabidopsis thaliana*, multiple protein sequence alignments were performed using the default parameters of ClustalW, based on the domain sequences of characterized LEA_2 proteins. The deduced amino acid sequences of LEA_2 domains from different subfamilies were manually refined using GeneDoc software and MEGA 11.0. MEME Online Applications were employed to identify conserved motifs in the protein sequences, with parameters set to an optimum motif width of 6–200 and a maximum of 10 motifs. Additionally, the conserved domain of the VfLEA_2 protein was analyzed using hmmscan and the NCBI Conserved Domain Database (CDD). Visualization of the phylogenetic tree, motifs and conserved domain analysis results for LEA_2 sequences in faba bean constructed using TBtools. And exon-intron structures were identified using TBtools software [51].

Promoter sequences spanning the 2000 bp upstream regions of the initiation codon (ATG) from 41 *VfLEA_2* genes were extracted from the faba bean genome. Cis-acting regulatory elements within these regions were predicted using the PLANTCARE online platform [52] (http://bioinformatics.psb.ugent.be/webtools/plantcare/html/) and visualized via TBtools software.

### Chromosome location and gene duplication analysis

To comprehensively elucidate the genomic distribution characteristics and evolutionary dynamics of the *LEA_2* gene family in faba bean, this study implemented a systematic analytical strategy: first, based on the physical map of the faba bean reference genome, all *VfLEA_2* genes were anchored to specific chromosomes using the Gene Location Visualize module in TBtools and accordingly named the *LEA_2* genes in faba bean. This generated high-resolution circos plots to delineate chromosomal localization, gene density and syntenic relationships. Subsequently, to detect genome-wide duplication events, the multiple collinearity scanning toolkit (MCScanX) was employed to systematically identify *VfLEA_2* duplication patterns, including tandem duplication, segmental duplication and whole-genome duplication (WGD) events, with default parameters to balance sensitivity and specificity. Furthermore, to investigate the evolutionary conservation of *VfLEA_2* genes across species, the Dual Synteny Plotter module in TBtools was utilized to perform pairwise genome collinearity comparisons between faba bean and six representative plant species: pea (*Pisum sativum*), grass pea (*Lathyrus sativus*), barrel medic (*Medicago truncatula*), red clover (*Trifolium pratense*), white clover (*Trifolium repens*) and hairy vetch (*Vicia villosa*). Homologous gene pairs were identified via syntenic networks, highlighting evolutionarily conserved blocks and putative functional diversification differentiation of *LEA_2* genes across species.

### Plant materials and stress treatments

The faba bean cultivar “Qinghai 13”, developed by the Northwest Institute of Plateau Biology, Chinese Academy of Sciences, was selected for this study. Uniform, disease-free seeds were cultivated in a climate-controlled greenhouse at Qinghai University. Seeds were soaked in distilled water for four days to promote germination and then transplanted into pots containing a peat-perlite mixture (2:1, v/v), with three plants per pot. They were grown under these conditions: a 16-hour light cycle (25 °C, 6000 lx) and an 8-hour dark cycle (20 °C).

Pods and seed tissues (excluding seed coats) were collected at 7, 14, 21, 28 and 35 days post-flowering. Root, stem, and leaf tissues were collected at 21 days. All tissues were immediately frozen in liquid nitrogen and stored at −80 °C.

To study the response of the *VfLEA_2* genes to abiotic stress, uniform 21-day-old seedlings were exposed to three plant hormone treatments: abscisic acid (ABA, 100 µM), methyl jasmonate (MeJA, 100 µM) and salicylic acid (SA, 100 µM); and three environmental abiotic stresses: low temperature (4 °C), salt stress (150 mM NaCl) and drought (20% w/v PEG6000). Distilled water was used as the control. Samples were taken at 0, 3, 6, 12 and 24 h after treatment, with three biological replicates per treatment.

### RNA extraction and expression analysis

Total RNA was isolated from 100 mg of homogenized tissue using the E.Z.N.A. Plant RNA Pro Kit (Omega Bio-Tek, USA). RNA integrity was verified via 1.5% agarose gel electrophoresis, while purity and concentration were measured using a NanoDrop 2000 spectrophotometer (Thermo Fisher Scientific, USA). First-strand cDNA synthesis was performed using the HiScript III RT SuperMix (Vazyme, China) in a 20 µL reaction volume.

Gene-specific primers for *LEA_2* family members were designed using Primer-BLAST (NCBI), with VfACTIN serving as the internal control [21, 53–57]. Quantitative reverse transcription PCR (qRT-PCR) was conducted using ChamQ SYBR Master Mix (Vazyme, China) on a QuantStudio 5 Real-Time PCR System (Applied Biosystems, USA). Each 20 µL reaction contained 1 µL cDNA template, 0.4 µM primers, and 10 µL SYBR mix. Relative gene expression was normalized to VfACTIN and calculated using the 2 − ΔΔCt method. Three technical replicates were included for each biological sample to ensure data accuracy. GraphPad Prism 10.4.1 (GraphPad Software, LLC, San Diego, California, USA) was used for data entry and statistical analyses, and to draw the bar graphs [21]. All gene expression bar graphs depict the mean ± SD from biological replicates. Statistical comparisons between groups were performed using unpaired two-tailed t-tests. *P*-values are annotated directly on the figures with asterisks (*p < 0.05, **p < 0.01, ***p < 0.001) [58].

## Supplementary Information

Supplementary Material 1. List of the 41 *VfLEA_2* genes identified in this study.

Supplementary Material 2. Analysis and distribution of conserved motifs in VfLEA_2 proteins.

Supplementary Material 3. Cis-regulatory elements in the promoter region of *VfLEA_2* genes in this study.

Supplementary Material 4. The 4 pairs of tandem duplicates in *VfLEA_2* genes.

Supplementary Material 5. The 8 pairs of segmental duplicates in *VfLEA_2* genes.

Supplementary Material 6. One-to-one orthologous relationships between faba bean and other six species.

Supplementary Material 7. Primer sequences for qPCR.

## Data Availability

All the data generated or analyzed during this study are included in this published article and its supplementary information files. The genomic DNA, coding sequences (CDS), protein sequences, and annotation files of faba bean were obtained from the NCBI database (https://www.ncbi.nlm.nih.gov/; BioProject Accession ID PRJEB52541). The corresponding data for Arabidopsis thaliana were retrieved from Arabidopsis database (https://www.arabidopsis.org/).
